# Curcumin enhances the anti-cancer efficacy of paclitaxel in ovarian cancer by regulating the miR-9-5p/BRCA1 axis

**DOI:** 10.3389/fphar.2022.1014933

**Published:** 2023-01-10

**Authors:** Yuwan Liu, Zhangjin Shen, Tingjia Zhu, Weiguo Lu, Yunfeng Fu

**Affiliations:** ^1^ Women’s Reproductive Health Laboratory of Zhejiang Province, Women’s Hospital, School of Medicine, Zhejiang University, Hangzhou, Zhejiang, China; ^2^ Department of Gynecologic Oncology, Women’s Hospital, School of Medicine, Zhejiang University, Hangzhou, Zhejiang, China

**Keywords:** curcumin, paclitaxel, ovarian cancer, miR-9-5p, BRCA1, synergy

## Abstract

**Background:** Patients with late-stage ovarian cancer still have a very poor prognosis due to chemotherapy resistance. Curcumin has been shown to synergistically enhance the therapeutic effects of multiple chemotherapeutic agents, but the potential involvement of curcumin in ovarian cancer is largely unknown. This study aimed to investigate whether curcumin has synergistic anti-cancer effects with paclitaxel in ovarian cancer and its underlying mechanism.

**Methods:** Ovarian cancer cell lines (SKOV3 and A2780) were treated with curcumin, alone or combined with paclitaxel. Cell viability, colony formation, EdU incorporation assays, and flow cytometry were used to assess cell proliferation, apoptosis, and cell cycle progression. The cytotoxic synergistic effect of curcumin and paclitaxel was detected by Calcusyn software. RNA immunoprecipitation assay was used to verify the interaction between miR-9-5p and BRCA1. qRT-PCR and Western blot were performed to detect gene and protein expression.

**Results:** We found that curcumin and paclitaxel synergistically inhibited proliferation and promoted apoptosis in ovarian cancer cells. Furthermore, curcumin and paclitaxel combination resulted in decreased miR-9-5p expression and increased BRCA1 expression. Functionally, miR-9-5p overexpression counteracted the synergistic effect of curcumin and paclitaxel on cell proliferation and apoptosis by targeting BRCA1. Meanwhile, in vivo experiments revealed that curcumin and paclitaxel combination dramatically suppressed the growth of transplanted tumors, while miR-9-5p mimics eliminated the growth inhibition of xenografts induced by the combined treatment.

**Conclusion:** Curcumin enhanced the anti-cancer efficacy of paclitaxel in ovarian cancer by regulating the miR-9-5p/BRCA1 axis. These findings provide strong evidence for clinical investigation of curcumin and paclitaxel combination as a novel strategy for ovarian cancer patients, and identify miR-9-5p and BRCA1 as key targets for regulating sensitivity to this therapy.

## Introduction

Ovarian cancer is the most lethal gynecological malignancy, and it is the leading cause of cancer-related mortality among women (Siegel et al., 2021). Despite some advances in treatment, the 5-year relative survival of patients with advanced ovarian cancer has not been dramatically improved during the past decades (Vaughan et al., 2011; Kuroki and Guntupalli, 2020). Paclitaxel (PTX), a member of the taxane class, is one of the most widely used antineoplastic agents, and is recommended as first-line treatment for many cancers, including ovarian cancer and breast cancer. The mechanism of action of PTX is to suppress the depolymerization of microtubules, causing an extended mitotic arrest that can lead to cell death (Long and Fairchild, 1994; Kavallaris, 2010). Combined PTX and platinum chemotherapy is recognized as the essential treatment, especially in advanced cases (Kuroki and Guntupalli, 2020). However, the continuous use of conventional cancer therapies leads to chemoresistance, and a large proportion of patients suffer disease recurrence with the development of chemoresistance. Chemoresistance is a tough problem, which ultimately causes ovarian cancer patients to face treatment failure and death (Pinato et al., 2013). While different targeted therapies, such as anti-angiogenic agents and PARP inhibitors, are showing bright prospects for persistent and recurrent diseases, they do not yet meet clinical demands. Therefore, developing new therapeutics is urgent for ovarian cancer patients.

Over the years, the concept of combination therapy has been introduced into the development of cancer treatment (Bayat Mokhtari et al., 2017). Intriguingly, Traditional Chinese Medicine has been widely used all over the world as a supplement and alternative therapy for various cancers. Curcumin (Cur), a natural phenolic compound derived from the Curcuma longa rhizomes, has comprehensive pharmacological properties, such as anti-inflammatory and anti-oxidant (Zhang et al., 2015; Su et al., 2016). Previous studies have shown that Cur can exert strong anti-cancer properties such as inhibiting cancer cell proliferation and promoting cancer cell death (Xu et al., 2021). Cur can also sensitize cancer cells to some chemotherapeutic drugs such as cisplatin and gemcitabine, so it can be useful in combination therapy in multiple cancers (Yallapu et al., 2010; Yoshida et al., 2017; Zhang et al., 2017; Zheng et al., 2021). Besides, Cur is listed as a “generally recognized as safe (GRAS)” compound by the FDA, supporting its safety and tolerability when co-administered with conventional chemotherapy (Gupta et al., 2013). Recently, several preclinical studies have shown that Cur enhanced PTX-mediated cytotoxicity in ovarian cancer cells and may be a promising drug to reverse multiple drug resistance in cancer therapies (Liu et al., 2016; Wei et al., 2017). However, the therapeutic effect of Cur and PTX combination in ovarian cancer and its underlying molecular mechanisms have not been fully revealed.

MicroRNAs (miRNAs) are single-stranded non-coding RNAs with approximately 22 nucleotides. miRNAs can participate in posttranslational modification by targeting the 3′untranslated regions (3′UTRs) of mRNA. miRNAs have been shown to be closely associated with tumorigenesis and tumor progression. miR-9-5p was recently implicated in cancers. Accumulating evidence suggests that miR-9-5p serves as an oncomiR, promoting cancer cell proliferation, invasion and migration in multiple cancers, such as non-small cell lung cancer and prostate cancer (Li et al., 2017; Chen et al., 2019). It has been reported that miR-9-5p also functions as an oncomiR in ovarian cancer. Sui et al. (2019) demonstrated that miR-9-5p, which is upregulated during the progression of ovarian cancer, could promote the malignant phenotype by sustaining the low levels of NF-κB1, Snail1, and CDH1 in ovarian cancer cells. Zhou et al. (2017a) proved that miR-9-5p has the ability to promote metastasis in ovarian cancer by directly targeting E-cadherin 3′-UTR. In addition, multiple lines of evidence have indicated that Cur could exert its anti-cancer properties *via* down/upregulation of a variety of miRNAs including miR-9 family (Sun et al., 2008). Cur-induced miR-9 expression significantly inhibited the proliferation and colony formation of oral squamous cell carcinoma cells (Xiao et al., 2014). These studies suggest that miR-9 family may be involved in the anti-cancer effects of Cur against ovarian cancer. However, whether miR-9-5p plays a role in Cur combined with conventional drugs in the treatment of ovarian cancer remains unclear.

In this study, the effects of Cur and PTX combination in the treatment of ovarian cancer and its underlying mechanism were explored. Our data supported that Cur and PTX combination synergistically suppressed the growth of ovarian cancer both *in vitro* and *in vivo*. We also reported for the first time that Cur and PTX combination decreased the expression of miR-9-5p, thus improving BRCA1 levels in ovarian cancer cells. Upregulation of miR-9-5p or knockdown of BRCA1 reversed the synergy between Cur and PTX on cellular proliferation and apoptosis. These findings suggest that miR-9-5p/BRCA1 axis is involved in the synergistic effect of Cur and PTX in ovarian cancer cells, which may help us to develop new treatment strategies for ovarian cancer.

## Materials and methods

### Cell culture and reagents

Human ovarian cancer cell lines SKOV3 and A2780 were purchased from the American Type Culture Collection (ATCC, United States). Ovarian cancer cell lines were cultured in McCoys’5 A or RPMI-1640 medium (BasalMedia, L110KJ), supplemented with 10% fetal bovine serum (Everyday Green, 13,011–8,611). All cells were maintained at 37°C in a 5% CO_2_ incubator. PTX (MedChemExpress, United States) and Cur (Sigma-Aldrich, United States) were dissolved in DMSO (Sigma-Aldrich, D2660) and diluted to appropriate concentrations in culture medium.

### Cell transfection

The miR-9-5p mimics and negative controls (miR-NC) were purchased from RiboBio (Guangzhou, China). BRCA1 siRNAs and negative controls (si-NC) were synthesized by GenePharma (Shanghai, China). BRCA1 plasmids were designed and synthesized by Genechem Biotech (Shanghai, China). miRNA mimics and siRNAs were transfected into ovarian cancer cells using DharmaFECT1 transfection reagent (Thermo, United States) as per manufacturer’s protocol. Transfection of plasmids was performed using X-treme GENE HP DNA Transfection Reagent (Roche, China) according to the manufacturer’s instructions. The target sequences of siRNAs were listed in Supplementary Table S1.

### RNA isolation and quantitative reverse transcription polymerase chain reaction (qRT-PCR)

The total RNAs were extracted from cells by using Trizol reagent (Invitrogen, United States). cDNA synthesis was performed using the PrimeScript™ RT reagent kit with genomic DNA (gDNA) Eraser (TaKaRa, Japan). qRT-PCR was performed using TB Green Premix Ex Taq Kit (Takara, Japan) to quantify the amount of miRNAs and mRNAs. GAPDH and U6 were used as internal controls. The relative mRNA and miRNA expressions were calculated by the 2 (^−ΔΔCt^) method. Primer sequences are presented in Supplementary Table S2.

### Western blot

Cells were lysed with RIPA buffer and PMSF (Thermo, United States). Then, proteins extracted from cells were separated using 10% SurePAGE gels (GenScript, United States) and transferred to PVDF membranes (Bio-Rad, United States). The membranes were incubated with specific primary antibodies overnight at 4°C, and then incubated with a secondary antibody for 2 h. Expression of GAPDH was regarded as an internal control. The protein bands were detected by using Image Quant LAS 4000 mini (ImageQuant LAS 4000 mini, United States). Primary antibodies used were listed in Supplementary Table S3.

### Cell viability assay and combination effect evaluation

SKOV3 and A2780 cells were seeded into 96-well plates with 4,000 cells per well and cultured overnight. Then, ovarian cancer cells were treated with the specific concentrations of PTX, Cur or their combination for 48 h. After drug treatment, the medium was removed, and then, CCK-8 solution (Dojindo Laboratories, Japan) was added to each well to detect the cell viability. After 2 h, the optical density (OD) values were measured at 450 nm by a spectrophotometer reader (Thermo, United States). The combination effect between PTX and Cur was quantified by Com-puSyn software (ComboSyn, Inc., Paramus, NJ, http://www.combosyn.com/feature.html) and determined by the combination index (CI). CI value <1 represented a synergistic effect.

### Cell proliferation and colony formation assay

For cell proliferation assays, cells were seeded into 96-well plates at a density of 2,000 cells per well and cultured overnight. Then, the specified concentrations of PTX, Cur or their combination was added to the cell culture medium. 10 μL CCK8 was added into the wells at 0, 24, 48, 72, and 96 h respectively, and incubated for 2 h. The OD values were measured by a spectrophotometer reader at 450 nm. For colony formation assays, 1,000 SKOV3 and A2780 cells were seeded into 6-well plates. After incubation overnight, PTX, Cur or their combination was added to the cell culture medium. 2.5 μM Cur and 2.5 nM PTX were used for SKOV3, while 2.5 μM Cur and 5 nM PTX were used for A2780. 2 weeks later, the colonies were washed with PBS, fixed with methanol and stained with .1% crystal violet solution. Cell colonies were imaged and counted.

### EdU incorporation assay

Briefly, SKOV3 and A2780 cells were seeded into 96-well plates at a density of 4,000 cells per well and incubated overnight. After treatment with PTX, Cur or their combination for 48h, the Cell-Light™ EdU Apollo567 *In Vitro* Kit (RiboBio, Guangzhou, China) was used to detect the EdU incorporation rate, according to the manufacturer’s instructions. The EdU incorporation rate was expressed as the ratio of EdU positive cells (red cells) to total Hoechst33342 positive cells (blue cells).

### Apoptosis assay

The Annexin V-FITC/PI Apoptosis Assay Kit (MultiSciences, China) was used to detect cellular apoptosis following the manufacturer’s instructions. Cells with appropriate density per well were incubated in 6-well plates for 24 h, followed by transfection and drug treatment for 48 h, the supernatant and cells were collected. Then, all cells were resuspended in 1 × binding buffer, stained with V-FITC and PI for 5 min in the dark. The apoptosis rate was detected by flow cytometry (BD Biosciences, United States).

### Cell cycle assay

Cell cycle progression was analyzed using a Cell Cycle Staining Kit (MultiSciences, China) according to the manufacturer’s instructions. Cells were collected and resuspended with a staining solution containing PI after Cur and/or PTX treatment for 48 h. The stained cells were analyzed by flow cytometry (BD Biosciences, United States).

### *In vivo* tumor assay

Female BALB/c nude mice, aged 4–6 weeks, were bought from Silaike Experiment Animal Company (Shanghai, China), and maintained in the animal research center of Zhejiang Chinese Medical University. The ovarian cancer xenograft tumors were established by subcutaneously injecting SKOV3 cells (1 × 10^7 cells in 100 μL PBS) into the back of mice. The tumor size was measured twice a week, and the tumor volume was calculated using the following formula: volume (mm^3^) = (width^2^ × length)/2. One week after cell inoculation, mice were randomly divided into groups with control and different treatments to ensure similar volume levels in every group. 21–28 days after cell inoculation, the mice were sacrificed and all tumor tissues were collected for further analysis.

### Immunohistochemistry (IHC)

Tumor tissues were fixed in 4% formaldehyde and embedded in paraffin for further assay. Paraffin tissue were cut into 4 μm sections. The sections were first dewaxed, hydrated, and antigenically repaired. Then, the sections were incubated in 3% H_2_O_2_ for 15 min at 37°C to inhibit endogenous peroxidase. Next, the sections were blocked using 10% goat serum at room temperature for 30 min, and then incubated with the primary antibody Ki-67 (1:200, ab16667, Abcam) overnight at 4°C. On the second day, the sections were incubated with the secondary antibody for 30 min, and then incubated with DAB and hematoxylin. Finally, tissue sections were photographed for analysis.

### RNA immunoprecipitation (RIP) assay

RIP assays were performed using a Magna RIP RNA Binding Protein Immunoprecipitation Kit (Millipore, MA, United States) according to the manufacturer’s protocols. Cells were lysed in complete RIP lysis buffer, then cell lysates were incubated with 5 μg anti-Argonaute 2 (AGO2) or anti-IgG antibodies overnight. Co-precipitated RNA was quantified by qRT-PCR.

### Statistical analysis

Statistical analyses were conducted with GraphPad Prism 8.0 (GraphPad Software, United States). Data were presented as the mean ± standard deviation (SD), and analyzed by Student’s t-test or ANOVA test. All experiments were independently repeated at least three times. When *p* < .05, differences were considered statistically significant.

## Results

### Cur and PTX exert synergistic cytotoxicity in ovarian cancer cell lines

**FIGURE 1:**
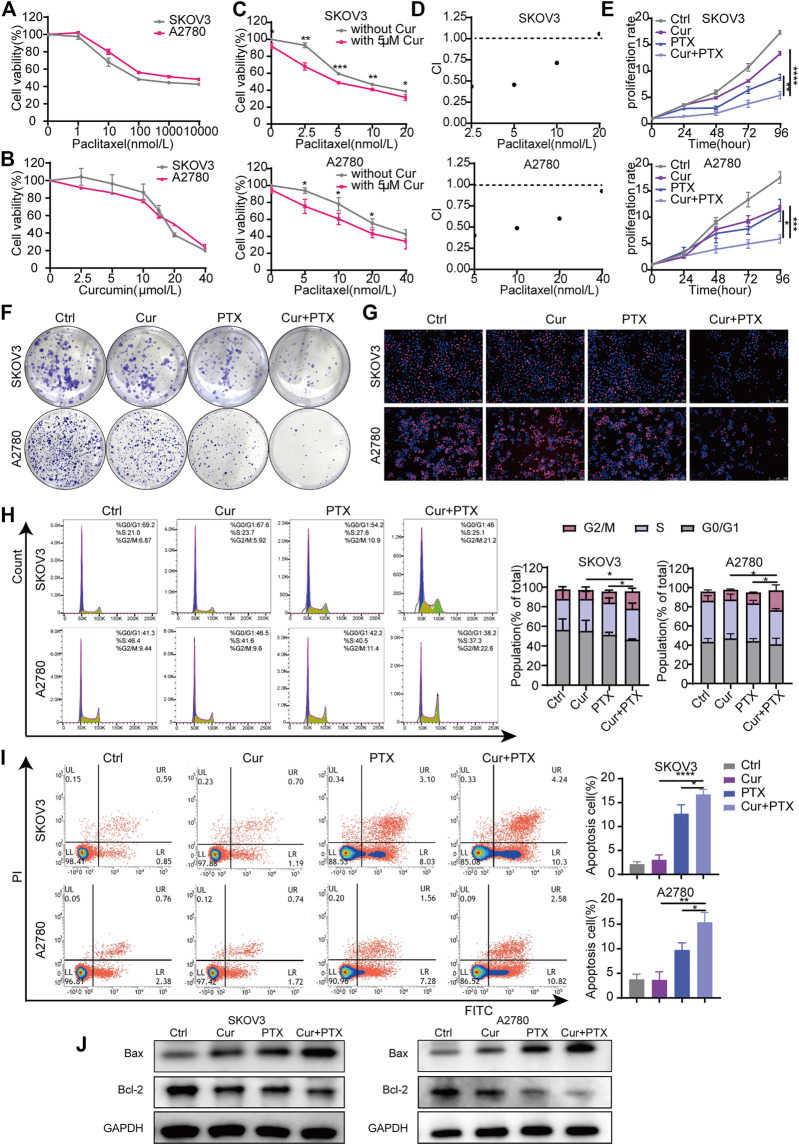
Cur and PTX exerts synergistic cytotoxicity in ovarian cancer cells. **(A,B)** Drug-response curves of survival after a series of concentrations treatment of PTX, or Cur in the ovarian cancer cell lines measured by CCK-8 assay at 48 h. **(C)** Dose–response curves of PTX with or without 5 μM Cur for the ovarian cancer cells. CCK-8 assays was used to determine cell viability. **(D)** The combination index (CI) of each combined treatment was calculated by using CalcuSyn software. CI > 1 indicates antagonist effect; CI = 1 indicates additive effect; CI < 1 indicates synergistic effect. **(E–G)** The proliferative capacity of ovarian cancer cells, treated with DMSO, Cur, PTX or their combination, were assessed by CCK-8 assays, colony-formation assays and Edu incorporation assays. **(H,I)** The cell cycle progression and apoptosis of ovarian cancer cells treated with DMSO, Cur, PTX or their combination for 48 h, detected by Flow cytometry.**(J)** The expression of Bax and Bcl-2 after indicated treatments monitored by Western blotting, GAPDH was used as a loading control. Data are representative of at least three independent experiments and presented as the mean ± SD. *p < .05, **p < .01, ***p < .001, ****p < .0001.

To evaluate Cur and PTX-induced cytotoxicity in ovarian cancer cells, the cell viability was measured by CCK8 assay in SKOV3 and A2780 cells after treatment with Cur or PTX alone. The results indicated that either Cur or PTX suppressed the viability of ovarian cancer cells in a dose-dependent manner (Figures 1A, B). Next, we selected a subtoxic concentration (5 μM) of Cur, which alone did not cause significant cytotoxicity, to evaluate its synergistic effect with PTX on ovarian cancer cells. The anti-cancer effects caused by Cur and PTX combination were significantly greater than that by a single agent in SKOV3 and A2780 cells (Figure 1C). Notably, except for the combination of 5 μM Cur and 20 nM PTX, the CI values of other groups in SKOV3 cells were less than 1. In A2780 cells, the CI values of Cur + PTX combinations were all less than one in all groups, and it indicated a strong synergy between Cur and PTX on ovarian cancer cells (Figure 1D). Then, 5 μM Cur and PTX (5 nM for SKOV3 cells and 10 nM for A2780 cells) were selected as the optimum concentration for further study.

To analyze the inhibitory effects of Cur, PTX or the combined treatment of both, cells were treated with the indicated concentrations, then CCK8, colony formation and EdU incorporation assays were performed. As shown in Figure 1E, compared with Cur or PTX alone, the combination of the two compounds obviously inhibited the growth of SKOV3 and A2780 cells. The colony formation assays showed a dramatic decrease of formed colonies in the combination groups, compared with single-agent groups (Figure 1F). Subsequently, EdU incorporation assays demonstraed that the number of EdU-positive cells was significantly decreased following treatment with Cur and PTX combination, compared with single drug treatment in each cell line (Figure 1G). Further cell cycle assays also indicated that the proliferative activities of cells were significantly inhibited after Cur and PTX treatment by inducing G2/M phase arrest (Figure 1H). Moreover, apoptosis assay were carried out to quantify the effects of Cur and PTX on cell apoptosis, and the results demonstrated that average apoptosis rates in the Cur-treated, PTX-treated, and (Cur + PTX)-treated SKOV3 cells were 3.02, 12.68, and 16.69%, respectively, indicating a synergistic cytotoxic effect of Cur and PTX. Similar synergistic cytotoxicity was also observed in A2780 cells (Figure 1I). Further, Western blot was used to detect the expression levels of apoptosis markers. Compared to DMSO controls, single-agent slightly increased the Bax level and decreased Bcl-2 level, but the Cur and PTX combination significantly increased the Bax level and decreased the Bcl-2 level in both SKOV3 and A2780 cells (Figure 1J).

Taken together, these data demonstrated that the combination of Cur and PTX exert synergistic cytotoxicity in SKOV3 and A2780 cells by inhibiting cell proliferation and promoting cell apoptosis.

### Cur and PTX synergistically suppress the tumor growth of SKOV3-derived xenograft models

**FIGURE 2:**
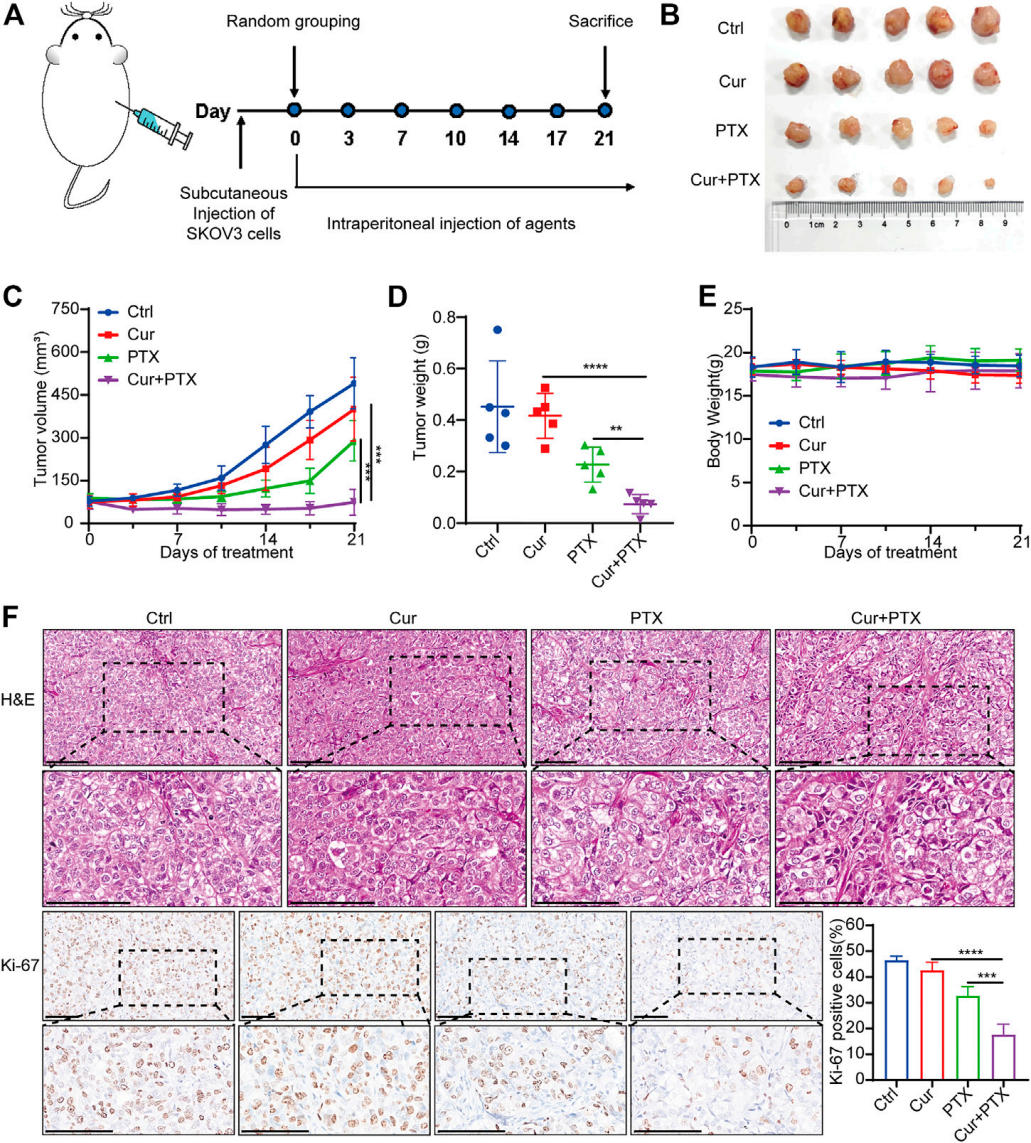
Cur and PTX synergistically suppress the tumor growth of SKOV3-derived xenograft models **(A)** Mice bearing SKOV3 xenografts were treated intraperitoneally with DMSO, Cur (20 mg/kg, B.I.W.), PTX (5 mg/kg/, B.I.W), or the combination of both agents as indicated (*n* = 5 mice per group). **(B)** Images of tumors collected from mice treated with DMSO, Cur, PTX or the combined agents. **(C,D)** Tumor volume and weight in the xenografts treated with different agents. **(E)** Mice weight growth curves after indicated treatments at different time points. **(F)** Representative images of H&E staining and IHC staining of Ki67 for xenograft tumor tissues after indicated treatments. Boxed regions were magnified and presented under the respective panels. Scale bar, 100 μm. Data are presented as the mean ± SD from five independent repeats. *p < .05, **p < .01, ***p < .001, ****p < .0001.

Based on the synergistic effect of Cur and PTX *in vitro*, we used a subcutaneous xenograft tumor model to evaluate the potential therapeutic effects of Cur and PTX *in vivo*. As shown in Figure 2A, tumor xenograft was established by subcutaneously injecting SKOV3 cells into nude mice. One week after cell inoculation, tumor-bearing mice were randomly assigned to four groups and intraperitoneally injected with Cur (20 mg/kg), PTX (5 mg/kg) or their combination twice a week. The results showed that the combination of Cur and PTX treatment caused markedly suppressed tumor growth (Figures 2B–D). Moreover, IHC analysis of Ki-67, a marker of cell proliferation, showed that the combination group showed the most obvious decrease in Ki-67 level (Figure 2F). These results highlighted the synergistic effects of the combined treatment of Cur and PTX *in vivo*.

To assess the biosafety of Cur and PTX combination treatment, changes in body weight and histological analysis were performed in mice. Notably, no obvious difference was found in the body weight of mice in the control or treatment groups (Figure 2E). Moreover, the administration of Cur and PTX combination did not cause any morphological changes in major organs (Supplementary Figure S1). These data demonstrated that the combination treatment was safe and well-tolerated.

### Overexpression of mir-9-5p eliminates the synergistic cytotoxicity between cur and PTX in ovarian cancer cell lines

**FIGURE 3:**
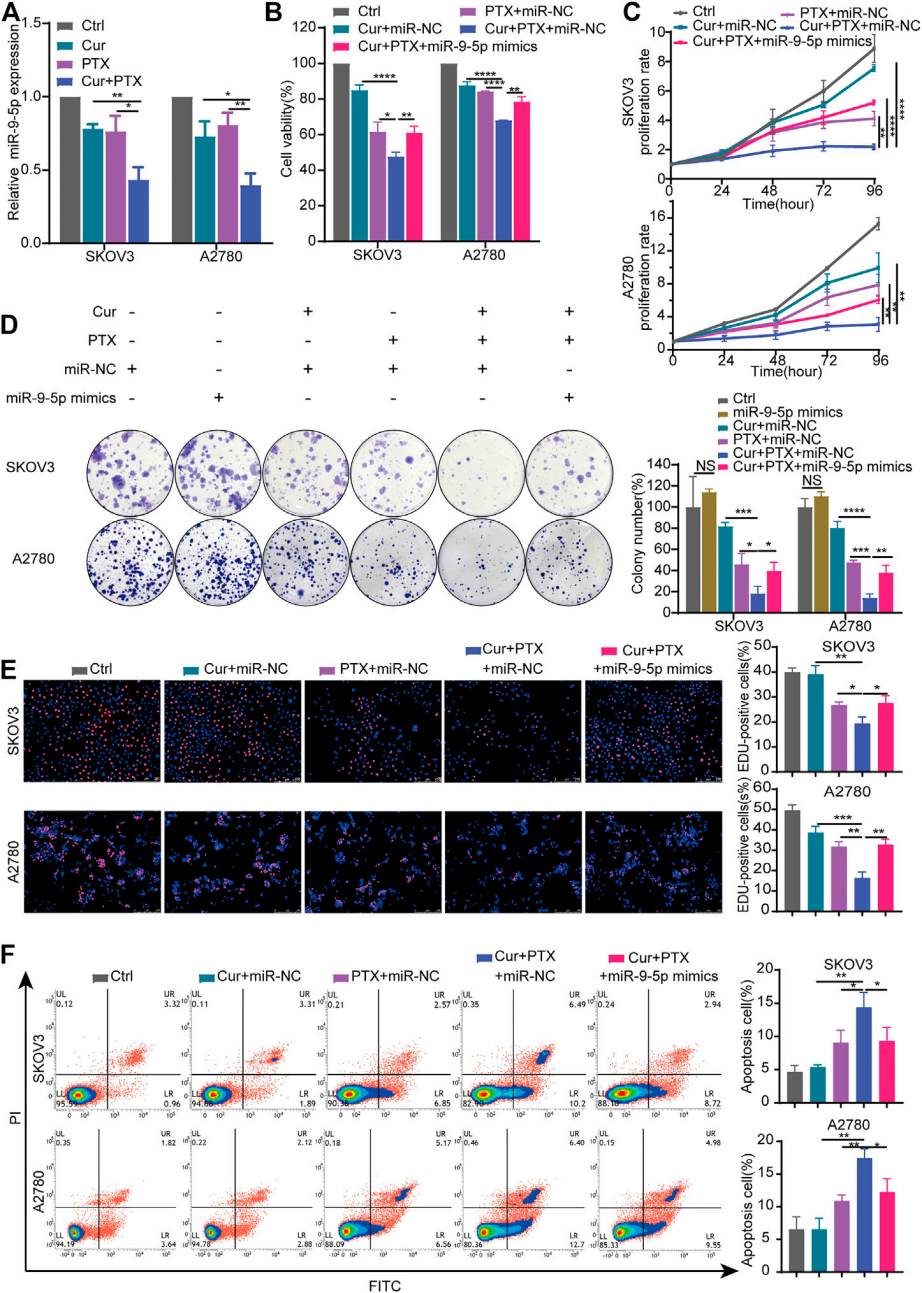
Overexpression of miR-9-5p counteracts the synergy between Cur and PTX in ovarian cancer cells. **(A)** The relative expression levels of miR-9-5p were detected by qRT-PCR in ovarian cancer cells treated with DMSO, Cur, PTX, or their combination. **(B)** Cell viability of SKOV3 and A2780 cells was detected by CCK-8 assays. Cells were treated with miR-NC or miR-9-5p mimics for 24 h, then combined treated with DMSO, Cur, PTX, or their combination for additional 48 h. **(C–E)** The proliferative capacity of SKOV3 and A2780 cells were assessed by CCK-8 assays, colony-formation assays and Edu incorporation assays. Cells were treated with miR-NC or miR-9-5p mimics for 24 h, then combined treated with DMSO, Cur, PTX, or their combination for the required time. **(F)** Cell apoptosis after indicated treatments was analyzed by Flow Cytometry. Data are representative of at least three independent experiments and presented as the mean ± SD. *p < .05, **p < .01, ***p < .001, ****p < .0001.

Cur has been reported to exert its therapeutic effects *via* regulating miR-9 expression (Mirzaei et al., 2016; Zhou et al., 2017b). Compared with the control, qRT-PCR revealed that Cur and PTX suppressed the miR-9-5p expression in SKOV3 cells or A2780 cells respectively. Moreover, the combination of Cur and PTX further decreased the miR-9-5p expression (Figure 3A). These results suggested that Cur and PTX may exert synergistic effects on ovarian cancer cells *via* targeting miR-9-5p.

To further explore the role of miR-9-5p in the synergy between Cur and PTX, we upregulated the miR-9-5p expression in SKOV3 cells and A2780 cells (Supplementary Figure S2), and then performed the CCK8, colony formation, EdU incorporation and apoptosis assays. As shown in Figure 3B, cell viability assay revealed that overexpression of miR-9-5p weakened the anti-cancer effects caused by Cur and PTX combination in SKOV3 and A2780 cells. In addition, compared with (Cur + PTX + miR-NC)-treated group, the proliferation rate, colony formation and EdU incorporation of ovarian cancer cells was significantly increased in the (Cur + PTX + miR-9-5p mimics)-treated group (Figures 3C–E). Next, the apoptosis assay demonstrated that Cur and PTX synergistically promoted the apoptosis of SKOV3 cells and A2780 cells, but overexpression of miR-9-5p eliminated this effect (Figure 3F). These results suggested that the synergistic effects between Cur and PTX was almost removed by upregulating miR-9-5p. That is to say, Cur and PTX exert synergistic effects in ovarian cancer *via* miR-9-5p.

### Overexpression of mir-9-5p eliminates the synergistic therapeutic effect between cur and PTX *in vivo*

**FIGURE 4:**
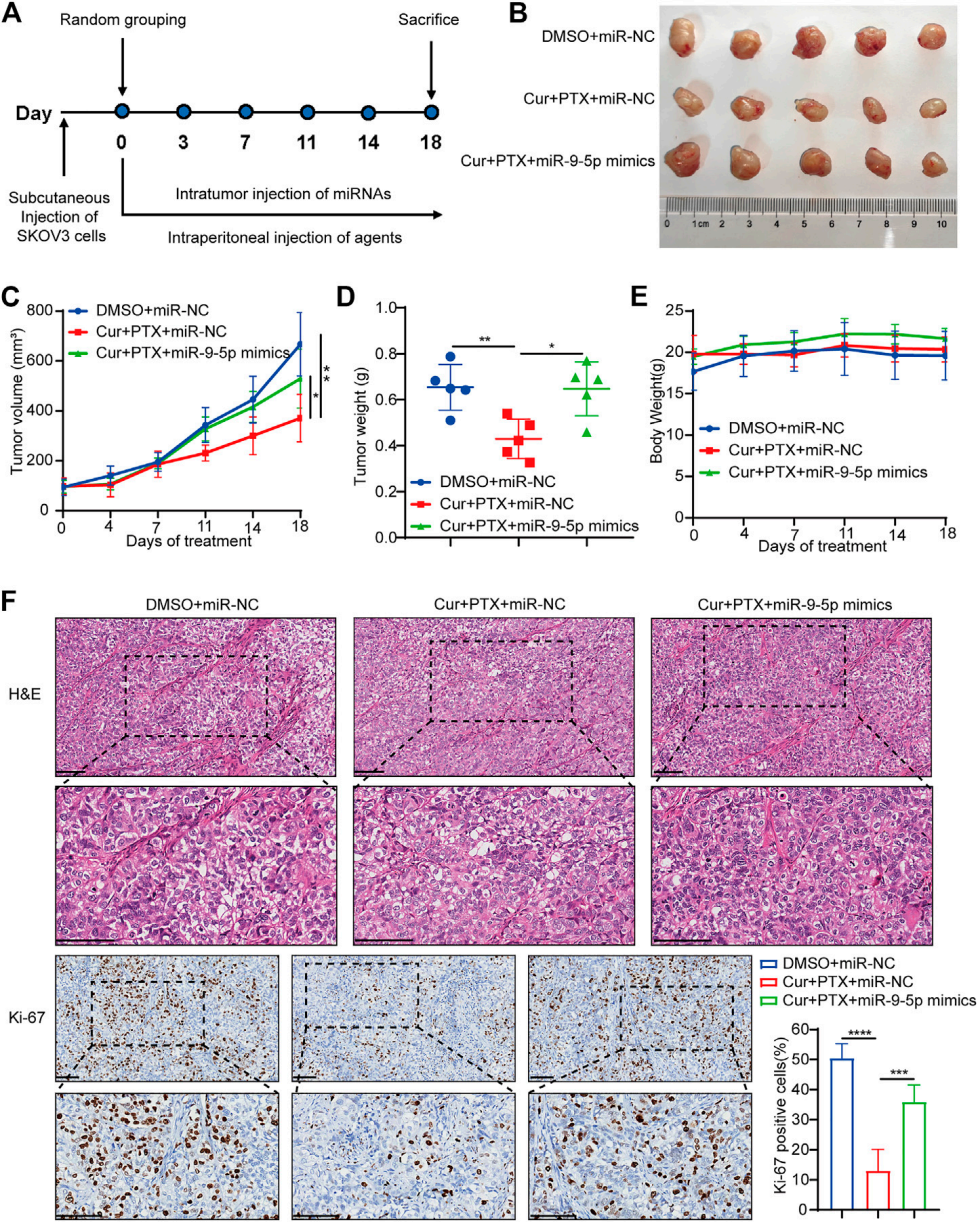
Overexpression of miR-9-5p counteracts the synergy between Cur and PTX *in vivo*. **(A)** Mice bearing SKOV3 xenografts were treated intratumorally with 50 nmol/kg miR-NC or miR-9-5p mimics, and combined treated with DMSO, or Cur and PTX combination. DMSO, Cur and PTX were administrated as mentioned above (*n* = 5 mice per group). **(B)** Images of tumors collected from mice treated with indicated agents. **(C,D)** Tumor volume and weight in the xenografts treated with different agents. **(E)** Mice weight growth curves after indicated treatments at different time points. **(F)** Representative images of H&E staining and IHC staining of Ki67 for xenograft tumor tissues after indicated treatments. Boxed regions were magnified and presented under the respective panels. Scale bar, 100 μm. Data are presented as the mean ± SD from five independent repeats. *p < .05, **p < .01, ***p < .001, ****p < .0001.

Next, we applied a subcutaneous xenograft tumor model to further validate the reverse of miR-9-5p on the synergistic effect between Cur and PTX *in vivo*. Tumor xenograft was established by injecting SKOV3 cells into the nude mice. As shown in Figure 4A, three groups of mice were treated as follows: 1) DMSO + miR-NC; 2) Cur + PTX + miR-NC; 3) Cur + PTX + miR-9-5p mimics. Cur and PTX combination dramatically inhibited the growth of tumors, while miR-9-5p mimics weakened the tumor-suppressive effects of combination treatment (Figures 4B, C). The tumor weight was significantly increased in the (Cur + PTX + miR-9-5p mimics)-treated group as compared with (Cur + PTX + miR-NC)-treated group (Figure 4D). Our data also indicated that these treatment protocols were safe as the mice behaved normally without obvious weight loss and toxicity in major organs (Figure 4E; Supplementary Figure S3). Moreover, IHC analysis showed a higher Ki67 level in the (Cur + PTX + miR-9-5p mimics)-treated group than in the (Cur + PTX + miR-NC)-treated group (Figure 4F). These results further confirmed the counteractive role of miR-9-5p on the synergistic therapeutic effects of Cur and PTX in ovarian cancer.

### miR-9-5p counteracts the synergy between cur and PTX in ovarian cancer cells by regulating BRCA1

**FIGURE 5:**
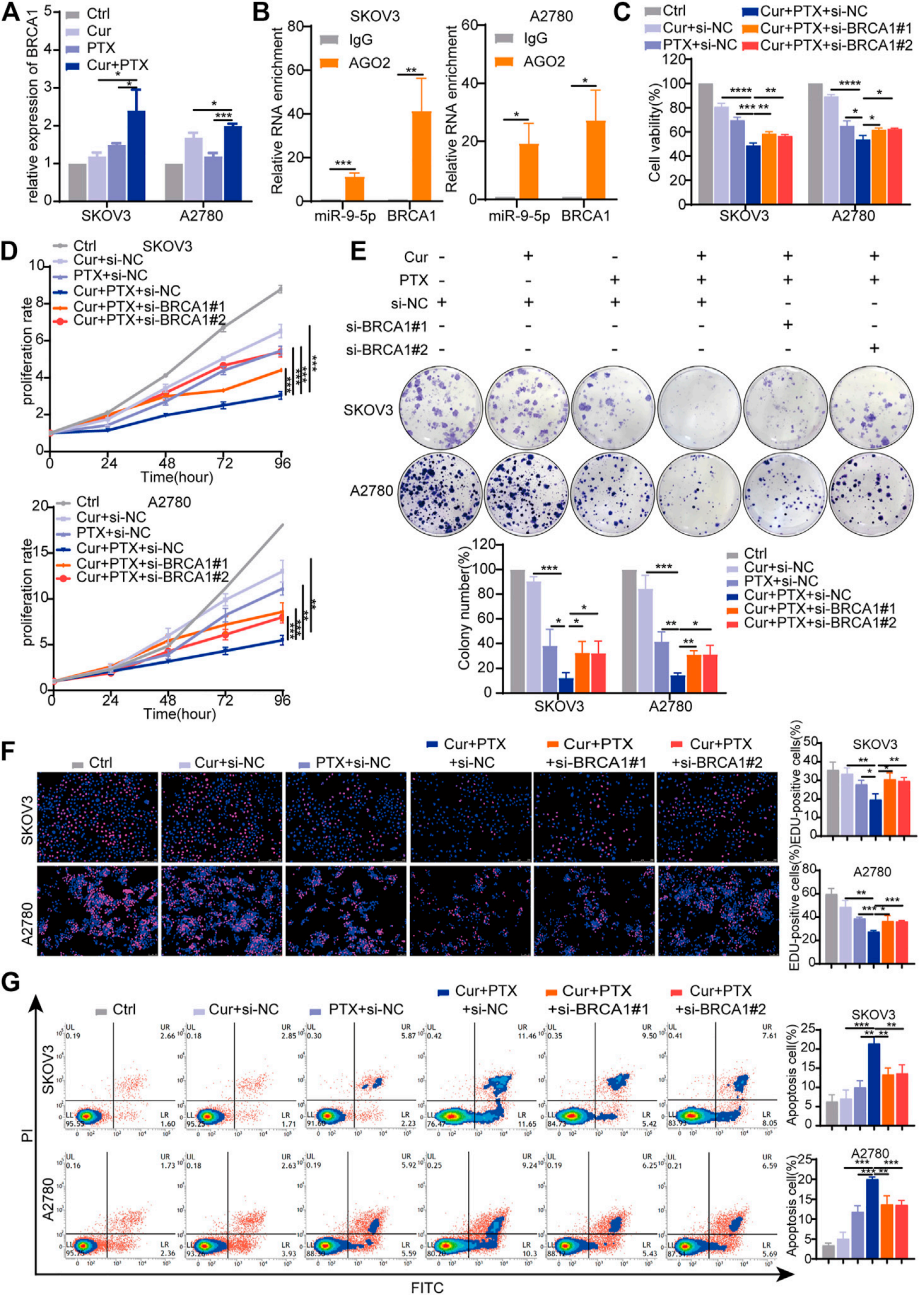
Knockdown of BRCA1 counteracts the synergy between Cur and PTX in ovarian cancer cells. **(A)** The relative expression levels of BRCA1 were detected by qRT-PCR in ovarian cancer cells treated with DMSO, Cur, PTX, or their combination. **(B)** The enrichment of miR-9-5p and BRCA1 in SKOV3 and A2780 cells was analyzed by anti-AGO2-RIP assays. **(C)** Cell viability of SKOV3 and A2780 cells was detected by CCK-8 assays. Cells were treated with siRNA for 24 h, then combined treated with DMSO, Cur, PTX, or their combination for additional 48 h. **(D–F)** The proliferative capacity of SKOV3 and A2780 cells were assessed by CCK-8 assays, colony-formation assays and Edu incorporation assays. Cells were treated with siRNA for 24 h, then combined treated with DMSO, Cur, PTX, or their combination for the required time. **(G)** Cell apoptosis after indicated treatments was analyzed by Flow Cytometry. Data are representative of at least three independent experiments and presented as the mean ± SD. *p < .05, **p < .01, ***p < .001, ****p < .0001.

BRCA1 was an experimentally verified target and its gene expression can be regulated by miR-9-5p (Sun et al., 2013a; Zhang et al., 2019; Zhang and Li, 2022). Previous studies have shown that BRCA1 is an important tumor suppressor gene responsible for DNA damage repair, and play complex roles in the occurrence and progression of ovarian cancer (King et al., 2003; Zhang and Powell, 2005; Kristeleit et al., 2022). Thus, we focused on BRCA1 in subsequent experiments, and proposed that BRCA1 has opposite effects on the synergy of Cur and PTX in ovarian cancer when compared with miR-9-5p. To test this, we applied qRT-PCR to assess the effects of Cur and PTX on BRCA1 levels, and found that BRCA1 expression was significantly upregulated in the combination groups, compared with groups treated with each drug alone (Figure 5A). The RIP results showed that compared with the IgG group, miR-9-5p and BRCA1 were abundantly enriched in the AGO2 antibody group (Figure 5B), further indicating that miR-9-5p degrades BRCA1 by binding to AGO2 protein and mediating RNA-induced silencing complex. Next, we applied BRCA1 siRNAs to ovarian cancer cells and then performed CCK8, colony formation, EdU incorporation and apoptosis assays. The knockdown efficiency in SKOV3 and A2780 cells was confirmed by qRT-PCR and Western blot (Supplementary Figures S4A, B). Our results found that, ovarian cancer cells treated with Cur + PTX + si-BRCA1 showed higher cell viability, proliferation rate, colony formation and EdU incorporation rate than cells treated with Cur + PTX + si-NC (Figures 5C–F). Besides, BRCA1 knockdown reduced cell apoptosis induced by Cur and PTX combination in SKOV3 cells and A2780 cells (Figure 5G). These results indicated that BRCA1 knockdown counteracted the synergistic effects between Cur and PTX on ovarian cancer cell lines.

**FIGURE 6:**
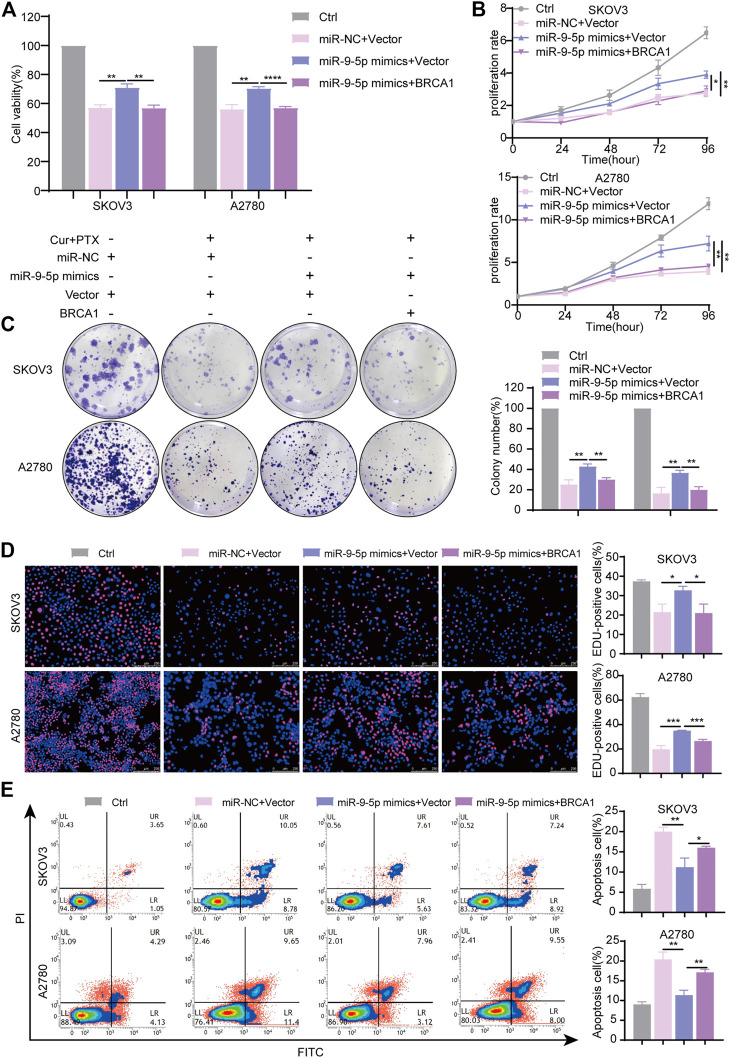
miR-9-5p counteracts the synergy between Cur and PTX by regulating BRCA1. SKOV3, and A2780 cells, transfected with miR-9-5p mimics, miR-9-5p mimics plus BRCA1 plasmid, and negative controls for 24 h respectively, were treated with DMSO, or Cur and PTX combination for a given time. **(A)** CCK-8 assays were performed to test cell viability. **(B–D)** CCK-8 assays, colony-formation assays and Edu incorporation assays were performed to detect the proliferative capacity of SKOV3 and A2780 cells. **(E)** Flow Cytometry were performed to analyse cell apoptosis. Data are representative of at least three independent experiments and presented as the mean ± SD. *p < .05, **p < .01, ***p < .001, ****p < .0001.

To further validate that miR-9-5p reverses the synergy between Cur and PTX *via* regulating BRCA1, a series of rescue experiments were performed using the same approaches as mentioned above. The results showed that BRCA1 plasmids significantly increased BRCA1 mRNA and protein levels (Supplementary Figures S5A, B). miR-9-5p mimics significantly revised inhibited cell viability induced by Cur and PTX combination, and BRCA1 plasmids eliminated the effects of miR-9-5p mimics (Figure 6A). Moreover, CCK8, colony formation and EdU incorporation assays proved that miR-9-5p mimics markedly revised inhibited cell proliferation induced by Cur and PTX combination, whereas BRCA1 overexpression counteracted this effect (Figures 6B–D). Consistent with the previous results, apoptosis assays indicated that miR-9-5p mimics dramatically reduced the apoptosis of SKOV3 and A2780 cells caused by Cur and PTX combination; however, the inhibited apoptosis induced by miR-9-5p mimics could also be partially eliminated after co-transfection with BRCA1 plasmids (Figure 6E). Taken together, all these data demonstrated that miR-9-5p functions through regulating BRCA1 in ovarian cancer cells.

## Discussion

Due to the frequent occurrence of chemoresistance, prognosis of ovarian cancer patients is not satisfactory (Siegel et al., 2021). Therefore, novel and potent treatments for ovarian cancer are still urgently required. In this study, we highlighted that Cur, in combination with PTX exerted synergistic cytotoxicity in ovarian cancer *in vitro* and *in vivo*. The *in vitro* experiments proved that Cur and PTX combination significantly inhibited cell proliferation and promoted cell apoptosis in ovarian cancer cells. Simultaneously, the *in vivo* experiments confirmed that this combination strategy showed synergistic anti-proliferation activity and satisfactory biosafety in mice bearing SKOV3 xenografts.

MiRNAs play crucial roles in the occurrence and development of multiple cancers. It has been reported that in head and neck squamous cell carcinoma, miR-9-5p could confer resistance to radiotherapy + cetuximab (Citron et al., 2021). Exosomal transfer of miR-9-5p augmented the resistance of breast cancer cells to tamoxifen by inhibiting ADIPOQ (Liu et al., 2021). Elevations of miR-9-5p in circulating extracellular vesicle may render resistance to docetaxel in breast cancer cells (Shen et al., 2019). In addition, previous studies have reported that Cur can regulate the expression of miR-9 family in cancer cells to exert its anti-cancer effect (Zhao et al., 2014; Mirzaei et al., 2016; Zhou et al., 2017b). Similar to the results reported previously, our study found that Cur caused a significant decrease of miR-9-5p expression in SKOV3 and A2780 cells. Notably, compared with Cur or PTX alone, Cur and PTX combination further suppressed the expression of miR-9-5p. These findings indicated that miR-9-5p may be involved in the synergy between Cur and PTX. Previous study demonstrated that miR-9-5p was upregulated in serous ovarian cancer, and overexpression of miR-9-5p could promote tumor metastasis *via* targeting E-cadherin (Zhou et al., 2017a). In the present study, we demonstrated, for the first time to our knowledge, that miR-9-5p mimics weakened the anti-cancer effects of Cur combined with PTX *in vitro* and *in vivo*, suggesting that overexpression of miR-9-5p counteracted the synergistic effect of Cur and PTX in ovarian cancer.

Several studies have shown that miR-9-5p could target the 3′UTR of BRCA1 (Sun et al., 2013b; Zhang and Li, 2022). It has been reported that BRCA1 is closely related to the occurrence of ovarian cancer, and its mutations are the main causes of ovarian cancer. Although women with BRCA1 mutation have an increased risk of developing ovarian cancer (Antoniou et al., 2003; King et al., 2003), the rate of BRCA1 mutation in ovarian cancer patients is only 10%–16% (Risch et al., 2006; Kwon et al., 2010). In addition, BRCA1 serves as an important tumor suppressor gene that controls recombination and transcription of chromatin (Zhang and Powell, 2005). In this study, ovarian cancer cell lines we used has wild-type BRCA1. We observed that BRCA1 expression in SKOV3 cells and A2780 cells was significantly upregulated in the combination treatment, compared to Cur or PTX alone. Therefore, we proposed a potential regulatory network between miR-9-5p and BRCA1 in the synergistic effects of Cur and PTX on ovarian cancer. A series of phenotypic experiments have supported this theory. The results suggested that BRCA1 knockdown counteracted the synergy between Cur and PTX in ovarian cancer cells. Moreover, the decreased synergistic effects of Cur and PTX caused by miR-9-5p mimics could be partially reversed after co-transfection with BRCA1 plasmids. Collectively, these results suggested that miR-9-5p/BRCA1 axis played a crucial role in the mechanism underlying the synergy between Cur and PTX in ovarian cancer.

In conclusion, results of the present study demonstrated that Cur, in combination with PTX exerted synergistic anti-cancer effect in ovarian cancer *in vitro* and *in vivo*. Mechanistically, miR-9-5p and BRCA1 may be potential biological targets involved in regulating the synergistic effect of Cur and PTX. Therefore, our novel findings suggest that Cur and PTX combination may be a promising strategy for ovarian cancer treatment, and targeting miR-9-5p/BRCA1 axis may help to improve therapeutic outcomes. However, further clinical studies are needed to evaluate the therapeutic efficacy of the combined treatment in ovarian cancer.

## Data Availability

The original contributions presented in the study are included in the article/Supplementary Material, further inquiries can be directed to the corresponding authors.
