# Supplementary material for: Curcumin enhances the anti-cancer efficacy of paclitaxel in ovarian cancer by regulating the miR-9-5p/BRCA1 axis
Source: Front Pharmacol. 2023 Jan 10;13:1014933. doi: 10.3389/fphar.2022.1014933 (PMC9871306; doi:10.3389/fphar.2022.1014933)
Supplement: Supplementary file 1 [file DataSheet1.pdf]

Supplemental materials

Table S1 Sequences of siRNAs against specific targets in this study.

Item	Sequence	
BRCA1 siRNA#1	Sense (5'-3')	GGGAUCUGAUUCUUCUGAATT
	Antisense (5'-3')	UUCAGAAGAAUCAGAUCCCTT
BRCA1 siRNA#2	Sense (5'-3')	CACCACAUCACUUUAACUATT
	Antisense (5'-3')	UAGUUAAAGUGAUGUGGUGTT
si-NC	Sense (5'-3')	UUCUCCGAACGUGUCACGUTT
	Antisense (5'-3')	ACGUGACACGUUCGGAGAATT

Table S2 Sequences of primers used for qRT-PCR in this study.

Item	Sequence	
BRCA1	Forward (5'-3')	ACCTTGGAACTGTGAGAACTCT
	Reverse (5'-3')	TCTTGATCTCCCACACTGCAATA
miR-9-5p	Forward (5'-3')	Designed by RiboBio (Guangzhou, China)
	RT (5'-3')	Designed by RiboBio (Guangzhou, China)
GAPDH	Forward (5'-3')	TCACCACCATGGAGAAGGC
	Reverse (5'-3')	GCTAAGCAGTTGGTGGTGCA
U6	Forward (5'-3')	CTCGCTTCGGCAGCACA
	RT (5'-3')	AACGCTTCACGAATTTGCGT

Table S3 Antibodies used in this study.

Antigens	Manufacturer	Application
BRCA1	Santa Cruz, USA	1:200 for WB
Bax	Abcam, USA	1:1000 for WB
Bcl-2	Santa Cruz, USA	1:200 for WB
GAPDH	Diagbio, China	1:1000 for WB

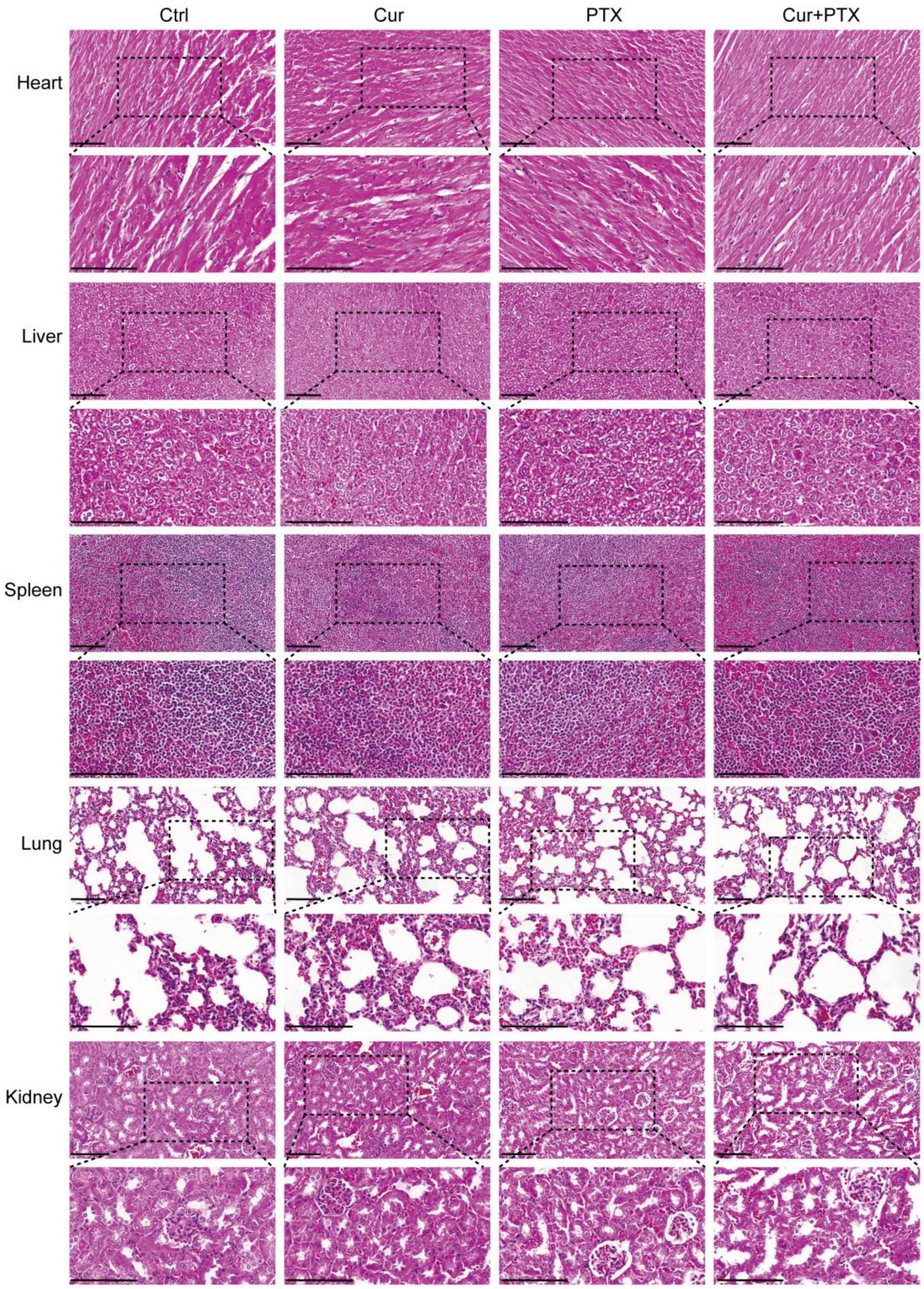

Figure S1. related to Figure 2.

H&E staining images of major organs (heart, liver, spleen, lung and kidney) from mice after different treatments (Scale bar = 100 μm).

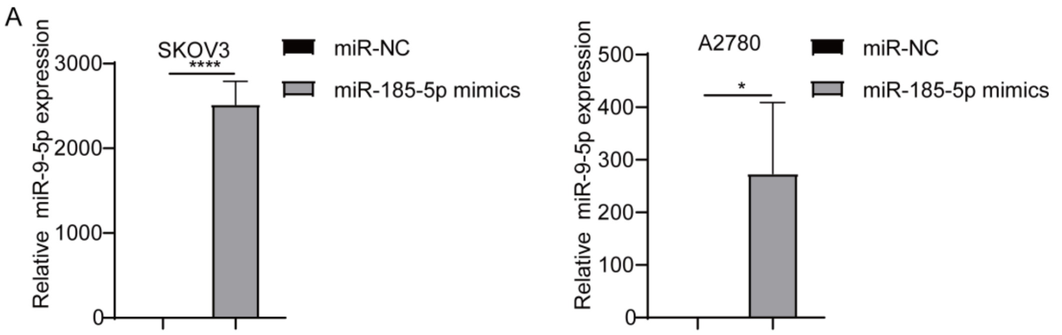

Figure S2. related to Figure 3.

The relative expression levels of miR-9-5p were detected by qRT-PCR in SKOV3 and A2780 cells transfected with miR-9-5p mimics or miR-NC.

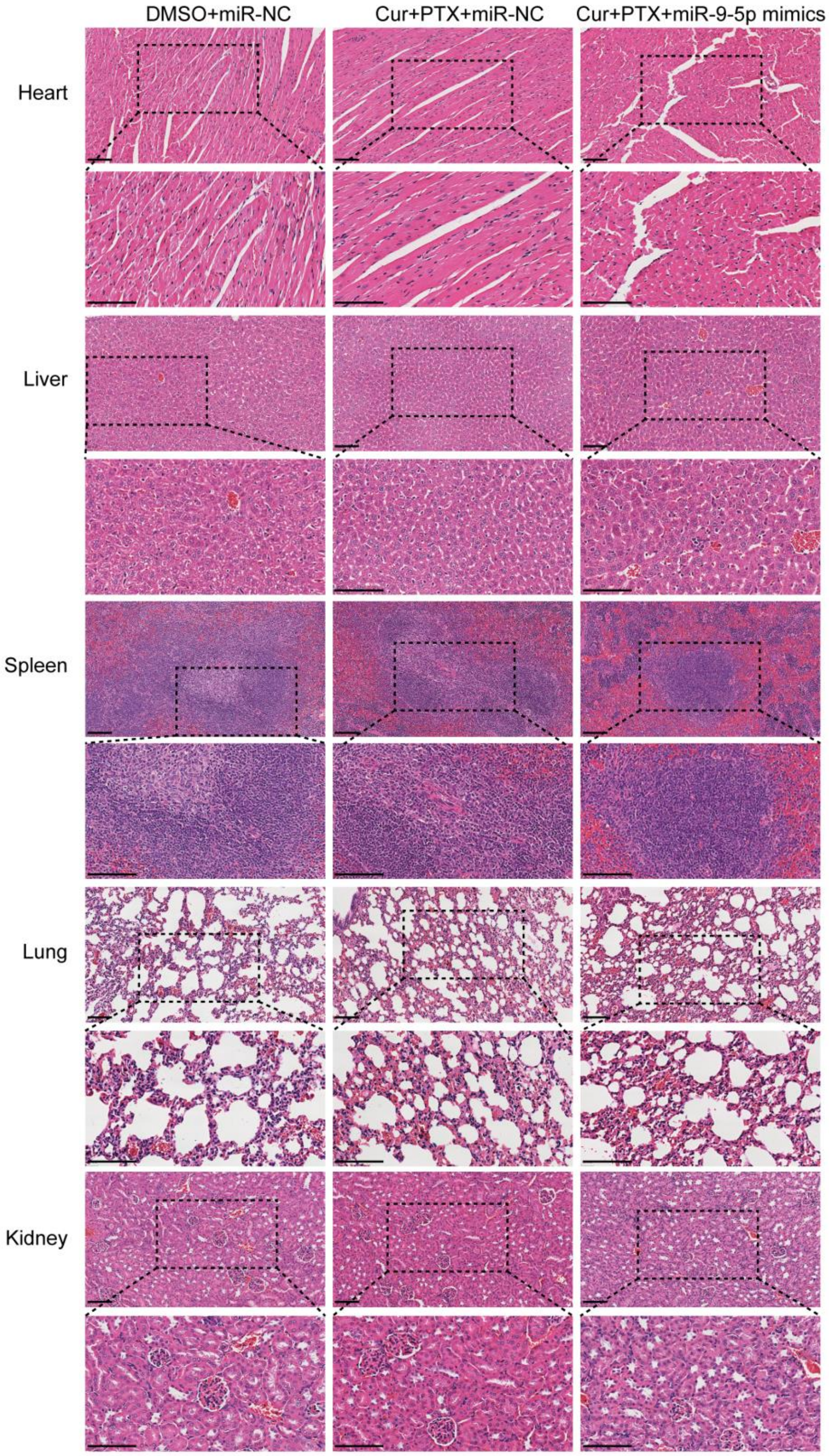

Figure S3. related to Figure 4.

H&E staining images of major organs (heart, liver, spleen, lung and kidney) from mice after different treatments (Scale bar = 100 μm).

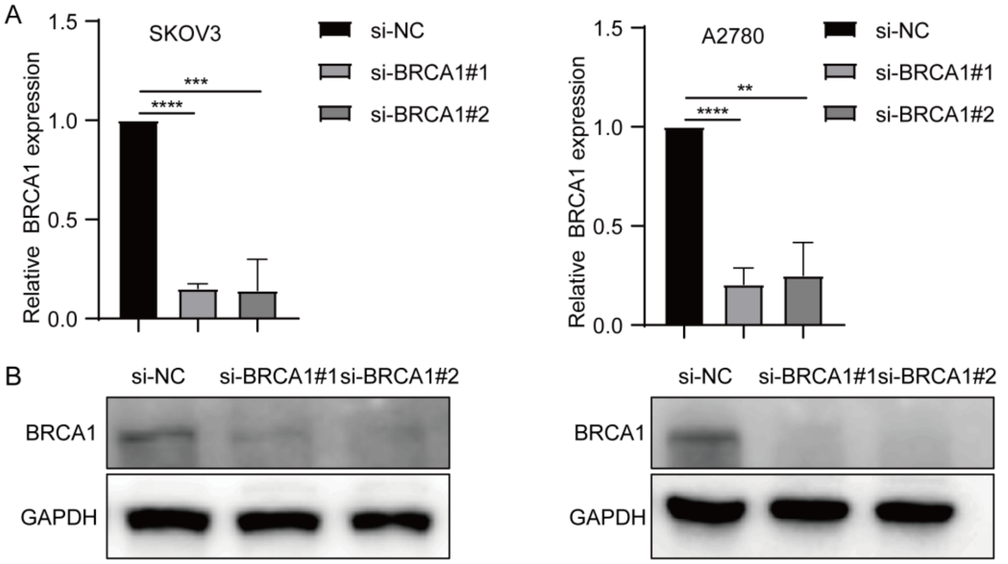

Figure S4. related to Figure 5.

A-B The relative levels of BRCA1 mRNA **(A)** and protein **(B)** expression in SKOV3 and A2780 cells transfected with si-BRCA1#1, si-BRCA1#2 or si-NC were detected by qRT-PCR and Western blotting, respectively.

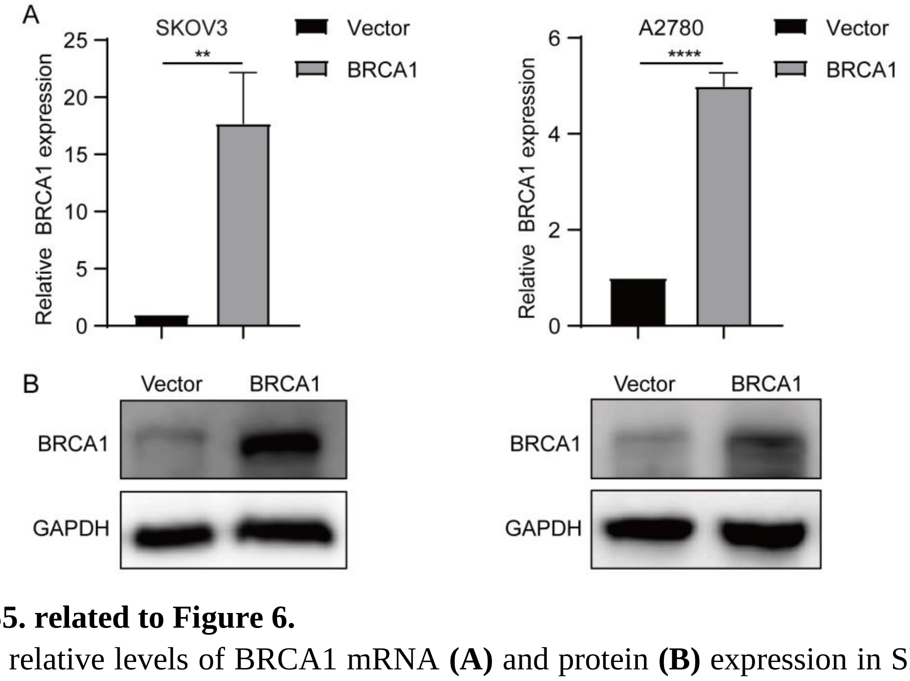

Figure S5. related to Figure 6.

A-B The relative levels of BRCA1 mRNA **(A)** and protein **(B)** expression in SKOV3 and A2780 cells transfected with BRCA1 plasmids or vector were detected by qRT-PCR and Western blotting, respectively.

Data are representative of at least three independent experiments and presented as the mean ± SD. *p < 0.05, **p < 0.01, ***p < 0.001, **** p < 0.0001.
